# Tips for teaching procedural skills

**DOI:** 10.1186/s12909-020-02284-1

**Published:** 2020-12-03

**Authors:** Annette Burgess, Christie van Diggele, Chris Roberts, Craig Mellis

**Affiliations:** 1 The University of Sydney, Faculty of Medicine and Health, Sydney Medical School - Education Office, The University of Sydney, Edward Ford Building A27, Sydney, NSW 2006 Australia; 2 The University of Sydney, Faculty of Medicine and Health, Sydney Health Professional Education Research Network, The University of Sydney, Sydney, Australia; 3 The University of Sydney, Faculty of Medicine and Health, The University of Sydney, Sydney, Australia; 4 The University of Sydney, Faculty of Medicine and Health, Sydney Medical School, Central Clinical School, The University of Sydney, Sydney, Australia

**Keywords:** Procedural skills teaching, Peyton’s four-step approach, Determining competency, Provision of feedback, Deliberate practice

## Abstract

The teaching of procedural skills required for clinical practice remains an ongoing challenge in healthcare education. Health professionals must be competent to perform a wide range of clinical skills, and are also regularly required to teach these clinical skills to their peers, junior staff, and students. Teaching of procedural skills through the use of frameworks, observation and provision of feedback, with opportunities for repeated practice assists in the learners’ acquisition and retention of skills. With a focus on the teaching of non-complex skills, this paper explores how skills are learned; ways to improve skill performance; determining competency; and the provision of effective feedback.

## Background

Health professionals must have the ability to perform a wide range of clinical skills competently. These generally include history taking, physical examination, and procedural skills. While some procedural skills are specific to particular disciplines, competency in the performance of skills is required to ensure the delivery of safe patient care. Examples include correct hand washing technique, gastric tube insertion, cannulation, resuscitation, correct use of crutches, bedside dysphagia assessment, bed-to-chair transfer, and gait analysis. A skill that is learned and retained beyond the period of practice, can be recalled and competently performed in a variety of clinical settings [1]. Health professionals are regularly required to teach these clinical skills to their peers, junior staff, and students. However, the effectiveness of skills teaching is uncertain, and there is evidence suggesting junior health professionals are overconfident in their ability to teach practical skills [2]. With a focus on non-complex procedural skills, this paper aims to explore how skills are learned; ways to improve skills performance; determining competency; and the provision of effective feedback.

### How are skills learned?

In the last half of the twentieth century, many motor learning theorists posited the required steps to teach a psychomotor skill [3–6]. Building on this work, researchers have since proposed motor learning models for teaching and learning procedural skills [7, 8]. Common to most skills teaching literature is that skills are best learned by following a sequenced and stepped approach to teaching – whether a simple or complex task. However, the majority of skills required in healthcare are complex, requiring more than seven skill elements [9], and are difficult to teach, learn and retain. It has been reported that when using George and Doto’s (2001) five-step model to teach a simple dental skill, novices were able to perform the task after one attempt [10]. Similarly it has been reported that use of Peyton's [7] four-step model enhanced medical students' aquisition of simple skills when learning suturing [11]. When teaching complex tasks, however, the four- and five- step models may have limited utility to assist skill acquisition and retention. For example, some studies have reported no difference in learning outcomes when using a two step, four step or five step approach to teaching complex skills, such as simulated manual defibrillation [12], laryngeal mask airway insertion [13], and simulated gastric tub insertion [14]. Nicholls et al. (2016), in their review of contemporary motor learning, suggest an integrated instructional model to teach multi-part psychomotor skills provides a more effective approach to teaching complex skills required for clinical practice. The authors have provided the educational steps required to teach complex psychomotor skills [9].

The method used for teaching skills differs from that for teaching content. Teaching of procedural skills utilising frameworks, observation and feedback, with opportunities for repeated practice assists in the learner's acquisition and retention of skills [7, 8, 15, 16]. Clinical skills, such as taking a patient history, performing a physical examination, synthesising and presenting data, require multiple cognitive and psychomotor skills. As such, clinical skills are more readily demonstrated than described. One of the difficulties is that once a skill is performed regularly – as an expert – it is performed subconsciously. As a result, it is not easy to break it down into structural steps to clearly communicate the process to others [2]. There are many ways to teach a skill, including the use of simulated patients, manikins, videos, virtual reality and computers. The use of procedural skills labs in teaching provides opportunities for safe practice before performing these procedures on patients. This gives learners the opportunity for practice, to receive immediate feedback, and to further refine their competence and confidence - before undertaking the procedure on a patient. Although some skills, such as resuscitation, can be taught in skills labs, students will generally need to practise carrying out safe procedures in the clinical setting. Generally, a skill:

- is learned, and not innate
- is able to be broken into explicit steps
- requires practice in order to improve
- has a specific goal or outcome, that is measurable

**Fig. 1:**
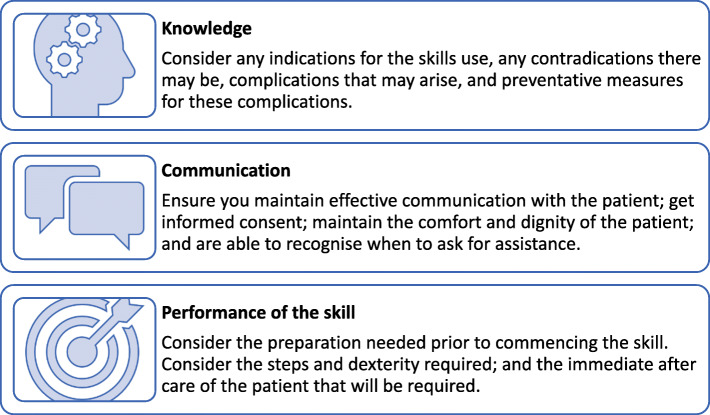
The three main components of skill competency [2, 17]

Learning a skill involves more than just performing the skill manually. There are other considerations, including knowledge of the procedure (such as why it is being done, how, and what are potential risks) and communication skills. Becoming competent in a skill involves three main components: knowledge, communication and performance [2, 17], as displayed in Fig. 1.

### Tips for teaching clinical skills

When teaching a skill, even the most basic of steps should be included, such as handwashing. No assumptions should be made, and every detail needs to be demonstrated. Some tips for teaching clinical skills are listed below [7]:

- *Include the fundamentals:* for example, handwashing
- *Demonstration:* provide clear demonstrations for learners to see
- *Integrate theory with practice:* learners can see the evidence behind the action, which promotes clinical reasoning
- *Break skills/procedures down into steps:* find out what the learners already know, and proceed from there
- *Use collaborative problem solving:* allow learners to work together towards a solution
- *Provide feedback:* that is clear and constructive, in an appropriate environment

### Skills as structural steps

Skills need to be broken down into small, discrete steps when teaching others in order to demonstrate and communicate exactly what is required. Although there are many models, a useful, well researched method is Peyton’s four step approach [7], displayed in Table 1, which can successfully be applied to teaching in the clinical setting. A controlled trial by Krautter et al. (2011) found that using Peyton’s four step approach to teach a technical skill was superior to standard instruction, with benefits in the areas of professionalism, communication and faster performance of the skill [14].

**Table 1:** Peyton’s four step approach to skills teaching [7]

Peyton’s four step approach	
1. ***Demonstration:*** Instructor demonstrates the skill at normal speed and without additional comments.	
2. ***Deconstruction:*** Instructor demonstrates the skill by breaking it down into simple steps, while describing each step.	
3. ***Formulation*:** Instructor demonstrates the skills whilst being ‘talked through’ the steps by the learner.	
4. ***Performance:*** Student demonstrates the skill, while describing each step.	

### Provision of immediate feedback

**Fig. 2:**
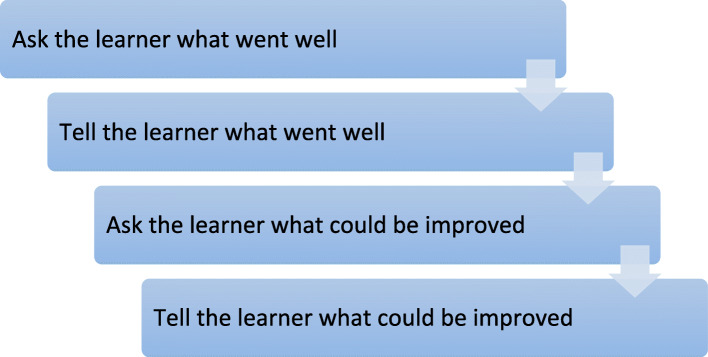
Feedback Model (adapted from Pendleton et al, 1984) [20]

The acquisition of procedural skills is reliant on task practice and feedback [2]. As well as repetition, Peyton’s four-step approach allows learners to see the skill being performed in real time, from beginning to end, and repeated by the instructor, before attempting performance of the skill themselves. This allows for the reinforcement of learning and opportunities to correct any errors and provide feedback. Immediate feedback and error correction avoids the risk of the skill being performed and practiced incorrectly, stored in long term memory, recalled and performed incorrectly [1, 18]. Provision of constructive feedback on the learners’ performance is an essential part of skills acquisition. Salmoni and colleagues (1984) suggest that feedback should be withheld until the conclusion of the skill to allow the learner to practice while focussing on each element of the skill, without excessive verbal information [19]. Feedback should be given immediately in order for the learner to correctly practice areas requiring improvement. The learner should also be provided with opportunities to ask questions at the end of the skill session. A participant-driven method, such as Pendleton’s feedback model [20], displayed in Fig. 2, is useful to ensure the learner reflects first on their own performance.

When training learners in skills teaching, Peyton’s four-step approach method [7], followed by provision of peer and teacher feedback can be modelled. For example, in small groups of three to five learners, each learner teaches a skill to another learner, using Peyton’s four-step approach. Each learner also takes responsibility for providing feedback to a peer on their teaching, using Pendleton’s feedback model. The activity (teaching a skill and providing feedback) may be formatively assessed by the facilitator using marking guides. However, it should be noted that as well as there being different models that may be used to teach a skill, there are also many models of feedback that can be applied to skills teaching. These include models such as Silverman’s SET-GO and ALOBA [21] methods, which can be usefully applied to bedside teaching. It is important to find suitable methods that you are comfortable and familiar with using.

### Development of competency

**Fig. 3:**
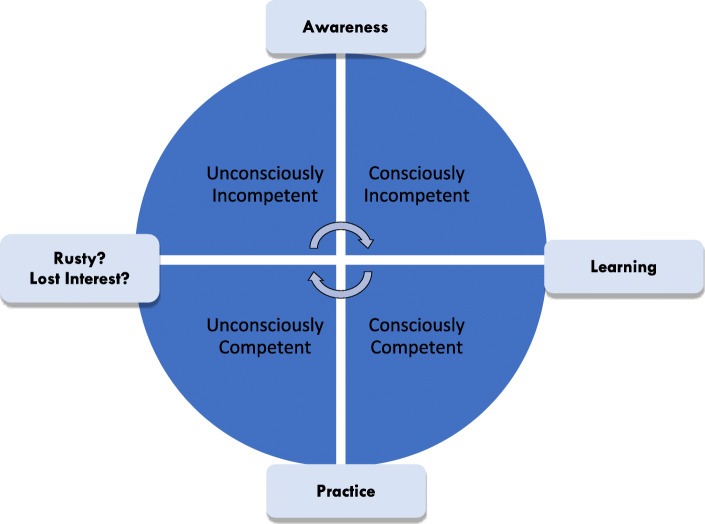
Development of Competency (adapted from Peyton, 1998) [22]

Learners move through a series of stages before becoming competent at a skill (see Fig. 3) [22]. There are four levels in skill acquisition: 1) Unconsciously incompetent, 2) Consciously incompetent, 3) Consciously competent, 4) Unconsciously competent. To appreciate these stages it is useful to reflect on acquisition of an everyday skill, for example, on how you learnt to drive a car. Novices will start initially as being ‘unconsciously incompetent’ (not aware of the knowledge and skills needed to competently drive), moving through the stages of competence (until they have the knowledge and skills to competently drive a car). However, there is the long-term potential of again becoming ‘unconsciously incompetent’ (for example, over-estimating their driving ability, and/or not staying up to date on new recommendations or road rules).

### Determining competency

Knowing when someone is competent can be difficult to assess. Learners are generally deemed competent once they can perform the procedure or skill alone, or without supervision. Competence is sometimes determined simply by noting the number of times the learner has performed the procedure (for example, bronchoscopy), or after completing a formal, observed assessment. Each skill may require a different approach to determining competence [23]. The important aspects of ensuring someone is competent are [23]:

- Setting and knowing the outcomes
- Setting and knowing expectations
- Multiple observations of skill performance
- Looking for common errors

**Fig. 4:**
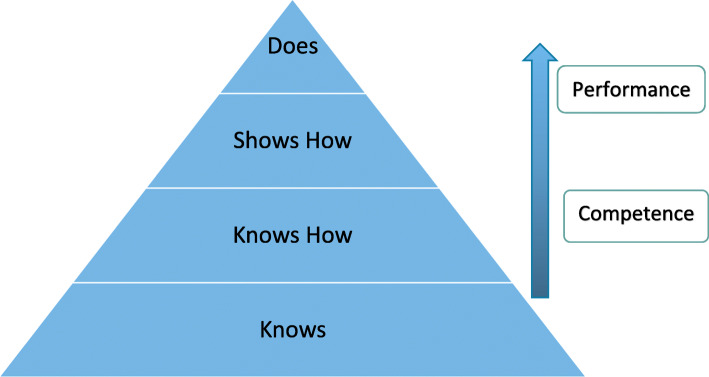
Framework for clinical assessment (adapted from Miller, 1990) [24]

Miller’s pyramid [24] provides a useful hierarchy to determine a learner’s competency in performing a skill (see Fig. 4). The bottom of the pyramid is based on knowledge (taught didactically and assessed by multiple choice questions (MCQs)), moving to ‘knows how’ (taught clinically and assessed with Objective Structured Clinical Examinations (OSCEs) or clinical long cases), to demonstrating how to carry out the skill by “on the job” performance. Most assessments that clinicians undertake are based on the upper levels of the pyramid and are carried out in the workplace.

### Maintaining a skill and improving performance

Skills are acquired through diligent practice [15]. Nicholls and colleagues (2016) suggest skill practice is reliant upon multiple, spaced, short duration, and variable tasks, with practice opportunities to promote skill acquisition and long-term retention by the learner [9]. In order to avoid natural skill decay, it is important for health professionals to maintain their skills once acquired. This can only be achieved through regular practice. According to Ericsson & Charness (2004), those that attain high skill levels do so because they continually reflect on their own performance [15]. They tend to focus on the areas in which they are not doing well and practice that competence. This is what is known as *deliberate practice* and is highly relevant for clinical skills training, as well as elite sportspersons and musicians. The key elements to deliberate practice are:

- Well defined tasks
- Opportunities to practice and improve
- Opportunities to repeat and reflect
- Regular feedback from an observer

### The use of simulation in teaching procedural skills

It is important to note that the use of simulation-based healthcare education has been associated with better patient care and improved patient safety [25]. The frequency and repetition with which a task is practiced impacts skill retention, recall, and transferability from the simulated to real clinical environment [1, 18]. Simulation offers the opportunity to practice procedures without any risk of patient harm, and is widely used as both a training and assessment tool. Goal-orientated learning using competency-based instruction, is a characteristic of adult learning. It reinforces a standard of training, rather than assuming that everyone who has been taught the skill can perform the skill. A recent systematic review showed strong evidence for the use of simulation and competency-based teaching paradigms in the effective teaching of procedural skills [26]. The systematic review found that the most effective approach in aligning procedural skills training with the needs of the adult learners is high-quality simulation that includes repetitive practice, mastery of learning, and deliberate practice, supplemented by visual aids, such as videos [26]. Sawyer et al. (2016) developed a six- step approach to teaching a skill that combines preparation, skill acquisition, and maintenance of the skill: “Learn, See, Practice, Prove, Do, Maintain” [25]. This six-step approach uses adult learning theory to reinforce the need for the development, assessment and maintenance of procedural skills:

1. Learn: knowledge acquisition
2. See: observation of the procedure
3. Practice: deliberate practice using simulation
4. Prove: competency is assessed
5. Do: the procedure is performed on a patient, with direct supervision until the learner is entrusted to perform the procedure independently
6. Maintain: continued clinical practice, supplemented by simulation-based training.

## Conclusion

Acquisition of competency in clinical and procedural skills is fundamental to healthcare training. In the clinical setting, there is a requirement to teach skills to others, so it is important to learn how to do this most effectively. Simulation offers the opportunity to practice procedures without any risk of patient harm, and is commonly used as both a training and assessment tool. It has long been posited that using a stepped structural approach best guides skills acquisition and retention. A framework, such as Peyton’s four-step approach [7], is a useful model to break the teaching of a skill into discrete steps. The provision of feedback is essential in reducing the gap between current and desired performance. Once skills are acquired, deliberate practice is important in maintaining and improving the performance of a skill.

### Take home message

**Table:** 

• Skills need to be broken down into smaller steps when teaching others, and the use of frameworks, for example, Peyton’s four steps provide useful approaches. • Provision of constructive feedback, for example, using Pendleton’s model is an integral part of the skills teaching process. • Once skills are acquired, they must be maintained through deliberate practice**.**	

## Data Availability

Not applicable.

## Abbreviations

MCQs: Multiple choice questions
OSCEs: Objective Structured Clinical Examinations
